# Impact of complement component 3/4/5 single nucleotide polymorphisms on renal transplant recipients with antibody-mediated rejection

**DOI:** 10.18632/oncotarget.21788

**Published:** 2017-10-10

**Authors:** Zijie Wang, Haiwei Yang, Miao Guo, Zhijian Han, Jun Tao, Hao Chen, Yuqiu Ge, Ke Wang, Ruoyun Tan, Ji-Fu Wei, Min Gu

**Affiliations:** ^1^ Department of Urology, Nanjing Medical University First Affiliated Hospital, Nanjing 210029, P.R. China; ^2^ Research Division of Clinical Pharmacology, Nanjing Medical University First Affiliated Hospital, Nanjing 210029, P.R. China; ^3^ School of Public Health, Nanjing Medical University, Nanjing 211166, P.R. China

**Keywords:** kidney transplantation, antibody-mediated rejection, complement, single nucleotide polymorphism, next-generation sequencing

## Abstract

Antibody-mediated rejection (ABMR) is an important risk of allograft dysfunction in kidney transplantation. The complement system is considered to be associated with the generation of alloreative antibodies and donor-specific antibodies. However, the association of complement single nucleotide polymorphisms (SNPs) with ABMR still remained unclear. Blood samples of 199 renal transplant recipients containing 68 with ABMR and 131 with stable graft function were collected, and analyzed by next-generation sequencing with an established gene panel. High quality readout was obtained in 18 *C3* SNPs, 9 *C4* SNPs and 22 *C5* SNPs. Concerning *C3* gene polymorphisms, after being adjusted with age, sex and immunosuppressive protocols, rs10411506 and rs2230205 were found to be statistically associated with ABMR in dominant model (rs10411506: OR=2.73, 95% CIs: 1.16, 6.68, *P*=0.028; rs2230205: OR=2.52, 95% CIs: 1.07, 5.92, *P*=0.034); rs10411506, rs2230205 and rs2230201 were found different in HET model (rs10411506: OR=3.05, 95% CIs: 1.22, 7.64, *P*=0.017; rs2230205: OR=2.90, 95% CIs: 1.20, 7.00, *P*=0.018; rs2230201: OR=2.41, 95% CIs: 1.03, 5.64, *P*=0.042). The linkage analysis showed relatively high linkage disequilibrium among these SNPs. In addition, no significant correlation was found between *C4* SNPs, or *C5* SNPs, and the development of ABMR. Our study firstly identified the two SNPs (rs10411506 and rs2230205) in *C3* gene were statistically correlated with ABMR in kidney transplantation. These findings may have implications for the diagnosis and prevention of ABMR.

## INTRODUCTION

Kidney transplantation is the optimal therapy for patients with end-stage renal disease [1]. Despite the advancement in novel immunosuppressive agents and surgical techniques, challenges still remain in the area of maintaining long-term stable allograft function and minimizing the rejection [2]. Among these, the prevention and treatment of antibody-mediated rejection (ABMR) plays a critical role, which has been emerged as an important cause of both short-term and long-term injury to transplanted kidney [3-5]. ABMR often occurs in the presence of alloreactive antibodies or donor-specific antibodies (DSAs) and leads to the deterioration in graft function [6]. Efficient measures, such as timely monitoring of alloreative antibodies, maintaining of adequate immunosuppressive agents, have been already taken. However, even with strict adherence, the development of ABMR still persists due to the lack of knowledge in its detailed mechanisms and the sufficiently noninvasive monitoring system for renal transplant recipients [7, 8].

ABMR, also known as humoral rejection, is an important cause of short-term and long-term graft injury. The latest Banff criteria for the diagnosis of ABMR include the following three components: detectable DSA, presence of *C4*d deposition and histological evidence, including vasculitis and glomerulonephritis [9]. In the pathogenesis of ABMR, endothelial tissue is a key target and damage to the graft is primarily attributable to antigen-antibody complex-mediated activation of the classical complement pathway, which triggers multiple downstream processes, such as the promotion of antigen presentation, recruitment of leukocytes and the promotion of inflammatory processes [10]. Moreover, activation of the complement system in solid organ transplantation often occurs in the acute period during the initial ischemia/reperfusion phase and the subsequent adaptive immune responses, contributing to the development of ABMR [11].

The complement dependent mechanism plays a vital role in the pathogenesis of ABMR [12]. The antigen-antibody complex on graft endothelium activates the classical complement pathway, inducing complement dependent cascade [9, 13]. The complement cascade leads to the formation of membrane attack complex (MAC) which disrupts the integrity of phospholipid bilayer of cells, killing the cells [9, 13]. Complement independent mechanisms such as antibody-cell-dependent cytotoxicity (ADCC) can also be mediated by antibodies [13]. Since most of the target antigens present on the endothelium, evidence of acute (glomerulitis, peritubular capillaritis) and chronic (transplant glomerulopathy) microcirculation injury can be found in the biopsy [13]. The damage of endothelium can also lead to the formation of microthrobus, degrading the function of allograft further [9]. In the complement system, all the three pathways, including the classical, alternative and lectin pathways, lead to the activation of *C3* component by *C3* convertases, release of *C3*b opsonin, *C5* converstion and eventually membrane attack complex *C5*b-9 formulation, which is the most critical step in the elaboration of the biological effects of the complement system [14-16]. Therefore, modulation of complement-associated reactions may well determine whether initial activation of the complement sequence eventuates in beneficial or detrimental effects for the recipients.

In recent years, some studies have focused on the influence of *C3* genetic polymorphisms on outcomes of kidney transplantation, and certain *C3* genotypes were identified. Among these, the role of two *C3*F allotypes, which are called *C3*F (fast) and *C3*S (slow), in the short-term and long-term allograft outcomes were the most genotypes reported so far. Mutations from glycine to arginine in a functional region (position 80) of *C3*F allotype could lead to the variant of *C3*S [17, 18]. This mutation is possibly associated with the ability of *C3* to interact with monocyte complement receptors [19]. As an indispensable part in classical activation pathway of the complement, the fourth complement component (*C4*) is important in the pathogenesis of ABMR in allograft [20]. *C4*d is a complement split protein without biological function formed during the *C4* activation and its thioester moiety enables *C4*d to bind endothelial cells and basement membrane with strong covalent bonding [13, 21]. Thus the detection of *C4*d on biopsy allograft tissues suggests the classical complement activation and the occurrence of ABMR [22]. In addition, complement 5 (*C5*) is a pivotal complement, which initiates the assembly of the membrane attack complex, and mediates chemotaxis of various immune cells [23]. The progression of complement activation from *C3* to *C5* results in a soluble cleavage product *C5*a, a highly potent chemoattractant and activator of neutrophils and monocytes [24]. Associated with ABMR, *C5*a down-regulates inhibitory FcγR and up-regulates activating FcγR by stimulating macrophages [25]. It is significant to realize that the potentially deleterious effects of the proinflammatory terminal complement component on endothelial cells are controlled by a variety of complement modulators, many of which act on the enzymatic components of *C3* and *C5* convertases [26]. Recently, a systematic assessment of gene polymorphisms in the complement system, including four *C3* allotypes (rs7951, rs11569450, rs11569523 and rs11672613), were performed to investigate the association with graft survival, serum creatinine, delayed graft function and acute rejection of kidney transplantation, and no significant outcome was found [27]. Moreover, previous studies demonstrate that certain genetic variants of *C5* are a risk factor for several immune related disorders [28, 29]. As a result, the complement system, containing *C3*, *C4* and *C5*, may play a crucial role in the development of ABMR episodes; on the other hand, the effects of complement-related single nucleotide polymorphisms (SNPs) still remained largely unknown.

Next-generation sequencing (NGS) technology is a powerful and cost-effective tool for large-scale DNA sequencing, which has already changed the way we think about scientific approaches in genetic and evolutionary research [30]. Compared to conventional method, the primary advantage of NGS technology is the inexpensive production of large volumes of sequence data. Currently, NGS has been applied to an increasing number of human diseases, such as tumors, kidney diseases and obesity [31-33].

In our study, by the application of NGS technologies and comprehensive literature review of *C3*/*C4*/*C5* genetic polymorphism-related studies, we designed to examine the association between reported *C3* SNPs, as well as *C4* and *C5* SNPs, and the occurrence of ABMR in kidney transplantation in a Chinese population.

## RESULTS

### Baseline characteristics of renal transplant recipients

The clinical characteristics of these 199 recipients are shown in Table 1. The total incidence of ABMR was 34.17% (68 out of 199 recipients). Between two renal transplant groups, there was no significant association of PRA and HLA mismatch. In addition, no statistical difference was observed in mean age, gender or immunosuppressive protocols. Among patients in ABMR groups, we further collected ABMR-related clinical information, such as C4d scoring, histological classifications and the level of serum DSAs, and reported them in Table 1. We did not observe any significant differences (*P*>0.05) in age, sex, donor type and immunosuppressive protocol between the stable and ABMR group.

**Table 1:** Comparison of baseline characteristics between ABMR and stable subjects

Characteristics	Stable group	ABMR group	*P* value
**Case number**	131	68	NS
**Age (years, mean ± SD)**	37.55±1.24	38.92±2.01	NS
**Male (%)**	62.60	55.88	NS
**Number of HLA mismatches**	3.52±0.83	3.41±0.76	NS
**PRA (%)**	0.00	0.00	-
***Immunosuppressive protocol***			NS
**Pred + MMF + CsA**	63	26	
**Pred + MMF + TAC**	59	34	
**Pred + MMF + CsA + SIR**	5	6	
**Pred + MMF + TAC + SIR**	4	2	
Type of ABMR^*^			
**Acute ABMR**	-	23	
**Chronic active ABMR**	-	45	
Grade of morphologic tissue injury^*^			
**Grade I**	-	25	
**Grade II**	-	33	
**Grade III**	-	10	
C4d Scroing by IF^*^			
**C4d1**	-	5	
**C4d2**	-	17	
**C4d3**	-	46	
***Criculating DSAs (MFI, mean ± SD)***			
**Class I**	-	1322.15 ± 545.82	
**Class II**	-	1185.22 ± 650.08	

ABMR, antibody-mediated rejection; NS, not significant; SD, standard deviation; HLA, human lymphocyte antigen; PRA, panel reactive antibody; Pred, prednisone; MMF, Mycophenolate Mofetil; CsA, Cyclosporin A; TAC, tacrolimus; SIR, sirolimus.

^*^The classification of ABMR are in accordance with Banff 2007 criteria.

### The association of *C3* genotypes and ABMR

A total of 18 reported *C3* SNPs were identified using the NGS technology. No deviation from HWE was observed for any *C3* polymorphism. The genotypic distributions of the determined *C3* polymorphisms in both groups are shown in (Supplementary Table 1).

**Figure 1:**
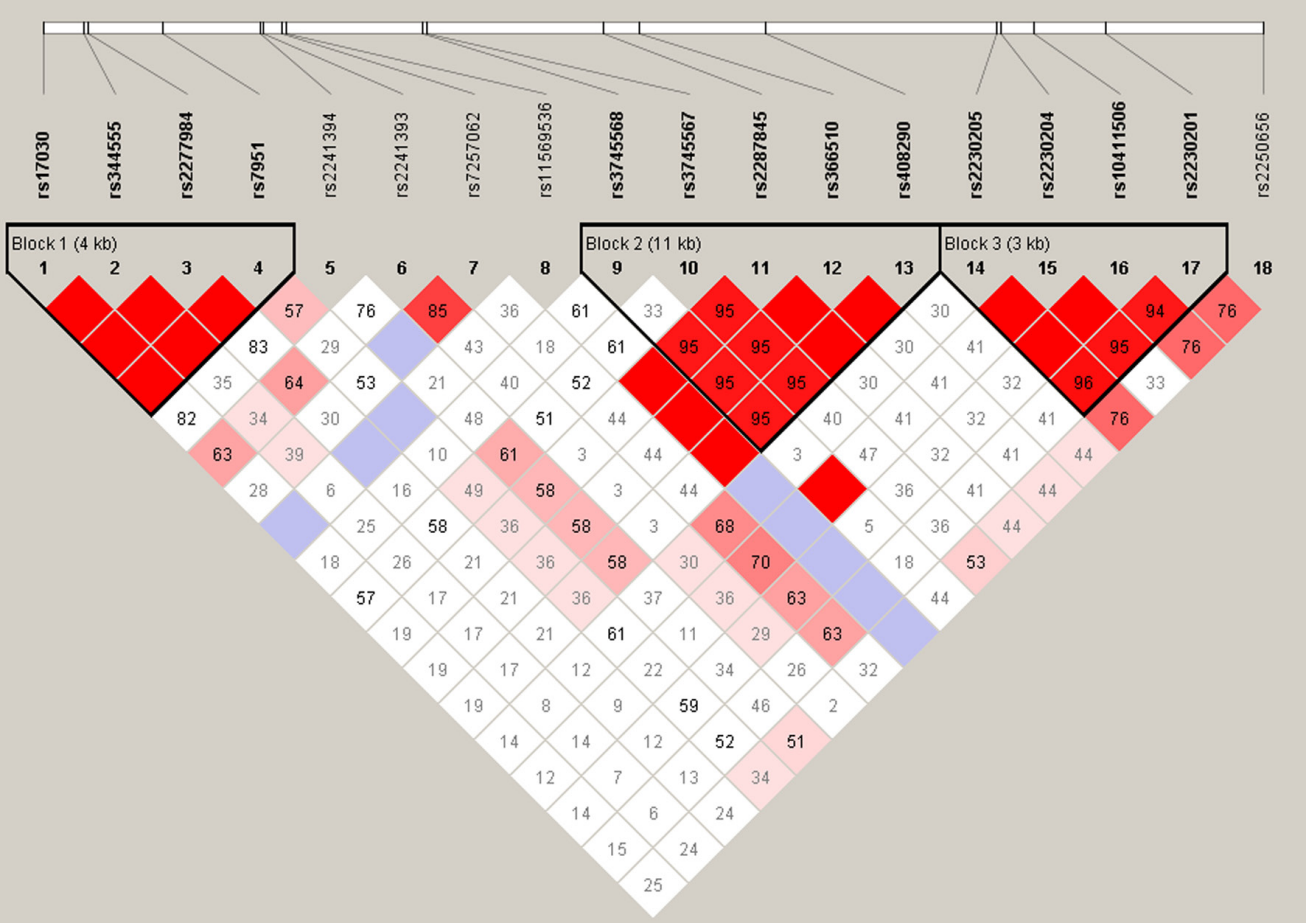
Linkage disequilibrium analysis of rs11569428, rs2230205, rs116528507, rs10411506, rs4807895 and rs2230201 in *C3* gene

A logistic regression analysis was performed after controlling for age, sex and immunosuppressive protocols as co-variables in all five analytical models (dominant, recessive, additive, HET and HOM) to explore the alternative effects of the variants. For dominant model, rs10411506 and rs2230205 were found to be significantly associated with the occurrence of ABMR [rs10411506 (GG vs. GA+AA): OR=2.73, 95% CIs: 1.16, 6.68, *P*=0.028; rs2230205 (CC vs. CT+TT): OR=2.52, 95% CIs: 1.07, 5.92, *P*=0.034; Table 2 ]. Moreover, for HET model, statistically significant difference was observed in rs10411506, rs2230205 and rs2230201 between two groups [rs10411506 (GG vs. GA): OR=3.05, 95% CIs: 1.22, 7.64, *P*=0.017; rs2230205: OR=2.90, 95% CIs: 1.20, 7.00, *P*=0.018; rs2230201 (CC vs. CT): OR=2.41, 95% CIs: 1.03, 5.64, *P*=0.042; Table 2 ]. In addition, there was no statistical difference between other SNPs in *C3* gene and the pathogenesis of ABMR (Table 2). Then, the SNPs of rs11569428, rs2230205, rs116528507, rs10411506, rs4807895, rs2230201 were tested for LD analysis, and the results indicate that these significant SNPs were in high LD status (Figure 1).

**Table 2:** Regression analysis for age-, sex- and immunosuppressive protocol-adjusted C3 genetic polymorphisms among recipients with ABMR

SNPs	Model	OR	95% CIs	*P* value
***rs17030***				
	Additive	1.14	0.74, 1.75	0.57
	Dominant	1.39	0.67, 2.89	0.38
	Recessive	1.02	0.51, 2.06	0.95
	HET	1.43	0.66, 3.10	0.36
	HOM	1.31	0.54, 3.16	0.55
***rs344555***				
	Additive	1.01	0.63, 1.63	0.96
	Dominant	1.26	0.68, 2.33	0.46
	Recessive	0.50	0.15, 1.65	0.25
	HET	1.44	0.76, 2.72	0.27
	HOM	0.60	0.17, 2.06	0.42
***rs2277984***				
	Additive	1.15	0.75, 1.78	0.52
	Dominant	1.39	0.67, 2.89	0.38
	Recessive	1.06	052, 2.14	0.87
	HET	1.41	0.65, 3.05	0.38
	HOM	1.34	0.55, 3.26	0.52
***rs7951***				
	Additive	0.97	0.47, 2.02	0.94
	Dominant	0.93	0.42, 2.08	0.86
	Recessive	1.61	0.095, 27.33	0.74
	HET	0.90	0.39, 2.06	0.80
	HOM	1.58	0.092, 26.84	0.75
***rs2241394***				
	Additive	1.40	0.53, 3.68	0.49
	Dominant	1.40	0.53, 3.68	0.49
***rs2241393***				
	Additive	0.96	0.34, 2.72	0.94
	Dominant	1.89	0.40, 8.94	0.42
***rs7257062***				
	Additive	1.03	0.42, 2.53	0.95
	Dominant	1.27	0.35, 4.63	0.72
	Recessive	0.61	0.061, 6.13	0.68
	HET	1.87	0.39, 9.05	0.44
	HOM	0.63	0.063, 6.27	0.69
***rs11569536***				
	Additive	1.34	0.11, 16.35	0.82
	Dominant	1.34	0.11, 16.35	0.82
***rs3745568***				
	Additive	1.48	0.64, 3.43	0.36
	Dominant	1.48	0.64, 3.43	0.36
***rs3745567***				
	Additive	1.45	0.57, 3.67	0.44
	Dominant	1.45	0.57, 3.67	0.44
***rs2287845***				
	Additive	1.68	0.88, 3.19	0.12
	Dominant	1.71	0.86, 3.39	0.13
	Recessive	2.68	0.15, 48.48	0.50
	HET	1.67	0.83, 3.35	0.15
	HOM	3.01	0.17, 54.73	0.46
***rs366510***				
	Additive	1.68	0.88, 3.19	0.12
	Dominant	1.71	0.86, 3.39	0.13
	Recessive	2.68	0.15, 48.48	0.50
	HET	1.67	0.83, 3.35	0.15
	HOM	3.01	0.17, 54.73	0.46
***rs408290***				
	Additive	1.18	0.80, 3.23	0.40
	Dominant	1.68	0.88, 3.23	0.12
	Recessive	0.93	0.42, 2.07	0.86
	**HET**	**2.91**	**1.19, 7.16**	**0.020**
	HOM	1.12	0.49, 2.54	0.79
***rs2230205***				
	Additive	1.26	0.79, 2.01	0.34
	**Dominant**	**2.52**	**1.07, 5.92**	**0.034**
	Recessive	0.078	0.36, 1.69	0.53
	**HET**	**2.90**	**1.20, 7.00**	**0.018**
	HOM	1.73	0.61, 4.88	0.30
***rs2230204***				
	Additive	1.08	0.68, 1.69	0.75
	Dominant	1.35	0.70, 2.61	0.37
	Recessive	0.76	0.31, 1.86	0.55
	HET	1.49	0.75, 2.97	0.26
	HOM	0.97	0.36, 2.59	0.94
***rs10411506***				
	Additive	1.32	0.82, 2.12	0.26
	**Dominant**	**2.73**	**1.12, 6.68**	**0.028**
	Recessive	0.85	0.40, 1.82	0.68
	**HET**	**3.05**	**1.22, 7.64**	**0.017**
	HOM	2.02	0.70, 5.86	0.20
***rs2230201***				
	Additive	1.23	0.77, 1.97	0.39
	Dominant	2.15	0.95, 4.90	0.068
	Recessive	0.82	0.38, 1.78	0.62
	**HET**	**2.41**	**1.03, 5.64**	**0.042**
	HOM	1.58	0.57, 4.36	0.38
***rs2250656***				
	Additive	1.31	0.75, 2.30	0.34
	Dominant	1.25	0.66, 2.34	0.49
	Recessive	2.75	0.43, 17.65	0.29
	HET	1.17	0.61, 2.23	0.64
	HOM	2.92	0.45, 19.02	0.26

SNPs, single nuclear polymorphisms; OR, odds ratio; CIs: confidential intervals.

### The association of *C4*/*C5* genotypes and ABMR

A total of 9 *C4* SNPs and 22 *C5* SNPs were identified. All genotype frequencies of stable group followed HWE. The genetic distributions of *C4/C5* SNPs screened in ABMR and stable subjects are shown in Supplementary Tables 2 and 3.

After adjusting the age, sex and immunosuppressive protocol, results of correlations between *C4* or *C5* SNPs and the development of ABMR were presented in Tables 3 and 4. Unfortunately, no significant association was found between the occurrence of ABMR and polymorphisms in *C4* or *C5* by applying various models.

**Table 3:** Regression analysis of *C4* genetic polymorphisms adjusted for age, sex and immunosuppressive protocols in ABMR and stable group

Position	Model	OR	95% CIs	*P* value
chr6:31963786				
	Additive	0.73	0.24, 2.27	0.59
	Dominant	0.77	0.23, 2.56	0.67
chr6:31964228				
	Additive	1.01	0.44, 2.36	0.97
	Dominant	1.17	0.46, 2.97	0.75
chr6:31964391				
	Additive	2.08	0.12, 36.64	0.62
	Dominant	2.08	0.12, 36.64	0.62
chr6:31964584				
	Additive	1.49	0.62, 3.59	0.38
	Dominant	1.49	0.62, 3.59	0.38
chr6:31994974				
	Additive	1.7	0.67, 4.31	0.26
	Dominant	1.7	0.67, 4.31	0.26
chr6:31996524				
	Additive	1.03	0.57, 1.86	0.94
	Dominant	1.30	0.65, 2.59	0.46
chr6:31996966				
	Additive	1.07	0.58, 1.99	0.83
	Dominant	1.03	0.51, 2.09	0.93
	Recessive	1.59	0.21, 12.02	0.65
	HOM	1.59	0.21, 12.15	0.65
	HET	1.00	0.48, 2.05	0.99
chr6:31997321				
	Additive	0.78	0.14, 4.29	0.78
	Dominant	0.78	0.14, 4.29	0.78
chr6:31997401				
	Additive	0.98	0.52, 1.82	0.94
	Dominant	1.01	0.53, 1.90	0.99

ABMR, antibody-mediated rejection; OR, odds ratio; CIs: confidential intervals.

**Table 4:** Regression analysis for age-, sex- and immunosuppressive protocol-adjusted *C5* genetic polymorphisms among recipients with ABMR

SNPs	Model	OR	95% CIs	*P* value
rs76339932				
	Additive	0.93	0.26, 3.27	0.90
	Dominant	0.93	0.26, 3.27	0.90
rs12237774				
	Additive	0.92	0.52, 1.63	0.78
	Dominant	0.92	0.47, 1.79	0.81
	Recessive	0.83	0.14, 4.89	0.83
	HET	0.94	0.47, 1.87	0.85
	HOM	0.81	0.13, 4.85	0.82
rs2300931				
	Additive	1.62	0.82, 3.15	0.16
	Dominant	1.59	0.74, 3.37	0.23
	Recessive	4.42	0.36, 53.99	0.24
	HET	1.44	0.65, 3.16	0.36
	HOM	4.69	0.38, 57.88	0.23
rs10985112				
	Additive	1.03	0.29, 3.64	0.96
	Dominant	1.03	0.29, 3.64	0.96
rs2269066				
	Additive	1.05	0.60, 1.81	0.88
	Dominant	1.12	0.59, 2.13	0.73
	Recessive	0.71	0.13, 3.94	0.69
	HET	1.17	0.60, 2.29	0.64
	HOM	0.74	0.13, 4.19	0.74
rs41260544				
	Additive	1.44	0.38, 5.44	0.59
	Dominant	1.44	0.38, 5.44	0.59
rs117287858				
	Additive	2.13	0.29, 15.88	0.46
	Dominant	2.13	0.29, 15.88	0.46
rs2230212				
	Additive	0.75	0.31, 1.78	0.51
	Dominant	0.75	0.31, 1.78	0.51
rs41311867				
	Additive	1.44	0.38, 5.44	0.59
	Dominant	1.44	0.38, 5.44	0.59
rs187517049				
	Additive	2.73	0.23, 31.84	0.42
	Dominant	2.73	0.23, 31.84	0.42
rs12683026				
	Additive	1.44	0.38, 5.44	0.59
	Dominant	1.44	0.38, 5.44	0.59
rs10985122				
	Additive	1.75	0.41, 7.47	0.45
	Dominant	1.75	0.41, 7.47	0.45
rs41309856				
	Additive	1.23	0.34, 4.44	0.75
	Dominant	1.23	0.34, 4.44	0.75
rs144465545				
	Additive	3.27	0.52, 20.59	0.21
	Dominant	3.27	0.52, 20.59	0.21
rs41309850				
	Additive	1.23	0.34, 4.44	0.75
	Dominant	1.23	0.34, 4.44	0.75
rs181763824				
	Additive	0.64	0.06, 6.55	0.71
	Dominant	0.64	0.06, 6.55	0.71
rs2230214				
	Additive	1.23	0.34, 4.44	0.75
	Dominant	1.23	0.34, 4.44	0.75
rs10985126				
	Additive	1.08	0.65, 1.77	0.77
	Dominant	1.15	0.62, 2.15	0.66
	Recessive	0.90	0.25, 3.20	0.87
	HET	1.19	0.62, 2.30	0.60
	HOM	0.96	0.26, 3.48	0.95
rs10985127				
	Additive	1.08	0.65, 1.77	0.77
	Dominant	1.15	0.62, 2.15	0.66
	Recessive	0.90	0.25, 3.20	0.87
	HET	1.19	0.62, 2.30	0.60
	HOM	0.96	0.26, 3.48	0.95
rs28426093				
	Additive	1.85	0.82, 4.15	0.14
	Dominant	1.57	0.59, 4.18	0.37
rs10818499				
	Additive	1.14	0.77, 1.70	0.51
	Dominant	1.54	0.81, 2.92	0.19
	Recessive	0.89	0.42, 1.85	0.75
	HET	1.81	0.89, 3.67	0.10
	HOM	1.19	0.52, 2.70	0.69
rs17216529				
	Additive	0.89	0.51, 1.54	0.67
	Dominant	0.94	0.49, 1.81	0.86
	Recessive	0.52	0.10, 2.78	0.45
	HET	1.02	0.52, 2.03	0.95
	HOM	0.53	0.10, 2.83	0.46

SNPs, single nuclear polymorphisms; OR, odds ratio; CIs: confidential intervals.

## DISCUSSION

In this study, we investigated the relationships between reported *C3* SNPs, as well as *C4* and *C5* SNPs, and the development of ABMR in renal transplant recipients. Our results showed that rs10411506, rs2230205 and rs2230201 located in *C3* gene, especially rs10411506 and rs2230205, were statistically associated with an increased risk of post-transplant ABMR following kidney transplantation. This is the first study to explore the presence and role of complement polymorphisms in ABMR after kidney transplantation.

As an important molecular in innate immune system, *C3* is the most abundant component of the complement pathways, which has a great impact on the downstream signals and activities [34]. *C3* component and its regulators are well recognized as the crucial factors in the susceptibility to immune-related diseases. Furthermore, multiple studies have shown that *C3* SNPs is associated with the pathogenesis of various diseases, such as age-related macular degeneration (AMD), ocular Behcet’s disease (BD), Vogt-Koyanagi-Harada syndrome (VKH) and chronic hepatitis C infection [28, 35, 36]. Among these various studies, the potential role of rs10411506 and rs2230205 were only studied in the pathogenesis of AMD in Chinese population, and the results showed no significant association of rs10411506 and rs2230205 with AMD [35]. However, our study showed that these two SNPs appeared to be an important risk of the ABMR in kidney transplantation. Moreover, recipients carrying with rs10411506 GG genotype were less susceptible to the occurrence of ABMR post-transplantation when compared with those with A allele. Similarly, the rs2230205 CC genotype was found to protect the recipients from experiencing ABMR. Besides, we also found that *C3* rs2230201 SNP was statistically associated with the development of ABMR in HET model, which was consistent with previous studies conducted in ocular BD and VKH syndrome, chronic hepatitis C infection and systemic lupus erythematosus [28, 36, 37]. Nevertheless, considering to the negative results of additive model, dominant model and recessive model, the relative relationship of rs2230201 SNP and ABMR in kidney transplantation by HET model was less convincing when compared with rs10411506 and rs2230205. The linkage analysis further identified the high LD among rs10411506, rs2230205 and rs2230201, which failed to perform the reconstruction of allotype analysis. Given that introns are usually several short sequences that regulate the expression of *C3*, the rs10411506 GG genotype and rs2230205 CC genotype may have essential impact on the regulation of *C3* protein, thus contributing to the relatively lower risk of ABMR in kidney transplantation [38].

In our study, we failed to observe the significant correlation between *C4*/*C5* SNPs and post-transplant ABMR. SNPs in *C4* were considered to be responsible for the differences between *C4A* and *C4B* isotypes, Rodgers and Chido antigenic determinants and to be associated with several autoimmune diseases [39]. A genome-wide association study conducted in healthy Chinese found eight SNPs resided in a 2-Mb MHC region on chromosome 6p21.3 region where RCCX module situates related to copy numbers of *C4* gene and one SNP (rs2857009) independently affected the concentration of *C4* level in serum [40]. Pertaining to correlations between *C4* variations and kidney transplantation, recent studies concentrated on gene copy number variations (CNVs) of *C4* and long term graft survival and suggested a possibly better prognosis in patients with low dose of *C4* gene [41]. The available studies indicate the possibility that *C4* SNPs have influence on CNVs of *C4* or directly affect the expression of *C4*, thus contributing to the potential regulation in classical complement pathway and changing the chance of ABMR occurrence.

Recently, JC Jeong et al. [23] carried out the systematic assessment of the complement gene polymorphisms, including seven SNPs in *C5* gene (rs12237774, rs2159776, rs17611, rs25681, rs2241004, rs10985126 and rs10818500) and one SNP (rs10404456) in the *C5*aR gene, on the kidney transplant outcomes, showing that the GGCG allotype of *C5* in both recipients and donors was associated with lower renal allograft, whereas *C5*aR genotypes of recipients were not associated with acute rejection, and there was also no statistically significant association between donor *C5*/*C5*aR genotypes and acute rejection function [42]. Importantly, this study focused on the long-term outcomes of renal transplant and acute rejection, instead of the subgroup analysis of ABMR from the acute rejection, which is more correlated with the activation and progress of the complement system. However, being restricted with the collected samples, we could not perform quantitative evaluation of serum *C5* in two groups to validate our outcomes in genetic polymorphisms, which requires further prospective research.

Recent study conducted by Ermini [27] focused on the influence of SNPs in complement system in the short-term and long-term outcomes of renal transplant, including delayed graft function, acute rejection, graft survival and serum creatinine, instead of the subgroup analysis of ABMR from the acute rejection, which is more correlated with the activation and progress of the complement system. Nevertheless, the case number of eligible recipients in ABMR and stable groups of our transplant center is limited, leading to the potential bias of our outcomes. Therefore, a large-scale, multi-center and well-designed study of the association of *C3* SNPs and ABMR in renal transplant recipients should be conducted in the future.

In summary, we show here for the first time that the rs10411506 and rs2230205 in *C3* gene are statistically correlated with the development of ABMR in renal transplant recipients, and no significant relationship of *C4* or *C5* SNPs were observed during the episodes of post-transplant ABMR. These findings may have implications for the diagnosis and prevention of ABMR, contributing to the promotion of the graft survival and patients’ life quality in kidney transplantation.

## MATERIALS AND METHODS

### Ethics statement

The study protocol was in accordance with the ethical standards of the Declarations of Helsinki and Istanbul. Being limited to the living-related transplantation of kidney tissues to their lineal or collateral relative not beyond the third degree of kinship or the cadaveric allograft donors of cardiac death (DCD), the protocol of this study was approved by the local Ethics Committee of the First Affiliated Hospital with Nanjing Medical University, and written informed consent was obtained from all transplant recipients. None of the transplant donors were from a vulnerable population, and all donors or next of kin freely provided written informed consent.

### Study design and subjects

#### Study design

This was a 12-month, retrospective, case-control trial containing 199 renal transplant recipients who underwent kidney transplantation between February 1^st^, 2008 and December 1^st^, 2015 in renal transplant center of the First Affiliated Hospital with Nanjing Medical University. This study was designed to investigate the distributions of *C3/C4/C5* SNPs between patients with period of ABMR and stable allograft function. The inclusion criteria to select the patients from stable group were as follows: [1]. The follow-up duration was longer than at least six months, and had never experienced the period of acute rejection, delayed graft dysfunction (DGF) or opportunistic infection; [2]. The concentration of serum creatinine (Scr) was lower than 120 μmol/L (1.36 mg/dl) for at least three months at the time of enrollment; [3]. Patients aged from 18 years old to 60 years old. Patients with following exclusive criteria were excluded in stable group: [1]. Patients aged less than 18 years old or older than 60 years old; [2]. History of acute rejection, DGF or opportunistic infection; [3]. Fluctuation of Scr over than 120 μmol/L (1.36 mg/dl) during the last three months of enrollment; [4]. Pregnant women and active HIV infection; [5]. Chronic lung disease requiring supplemental oxygen therapy. To enroll patients into the ABMR group, patients with significant clinical characteristics, such as an increase in serum creatinine level by 20% from baseline (not attributable to other cases) and overloaded urine protein, were required to perform the indication allograft biopsy immediately before the administration of high-dose steroids therapy. The diagnostic criteria of ABMR were mainly based on the comprehensive histological examination according to Banff 07 classification [43].

#### Data collection

Medical records were critically reviewed and related data, including age, gender, transplant date, duration of transplantation, transplant times, immunosuppressive protocol, were extracted by at least two clinicians for patient selection. Data on panel reactive antibody (PRA) and human leukocyte antigen (HLA) mismatch during pre-transplant period were also collected.

#### Subjects

Intravenous infusion of 500 mg/d of methylprednisolone was used during the surgery and up until 2 days after the operation. Then the dosage was reduced to 400 mg, 300 mg, 200 mg and 80 mg each subsequent day, followed by prednisone 30 mg/d as a maintenance therapy. In addition, Basiliximab (20 mg) was intravenously used at 30 minutes before the operation and the fourth day after the operation, respectively. All recipients received a three-drug or four-drug immunosuppressive regimen: Cyclosporin A (CsA) (n=100) or tacrolimus (n=99) in combination with mycophenolate mofetil (MMF) and prednisone, with or without sirolimus (n=17). The dosage of CsA and tacrolimus was started at 8 mg/kg/d and 0.2mg/kg/d, respectively, and then adjusted according to results of therapeutic drug monitoring the serum creatinine levels. A dosage of 200 mg/d of intravenous methylprednisolone was adopted for ABMR episodes with three to five days.

### Sample collection, preparation and NGS

Peripheral blood samples (2ml) from each recipient included in our study were collected with BD Vacutainer tubes containing sodium heparin when they were admitted to our center before the renal biopsy. Then, each collected blood sample was immediately transferred to the laboratory and stored at -80°C. The DNA of subjects was extracted from collected peripheral blood samples using QIAmp DNA Mini Kit (Qiagen, Hilden, Germany). Quantitative detection of concentration and purity of genomic DNA (gDNA) was performed by NanoDrop ND2000 (Thermo, MA, USA), while the gene integrity was tested by agarose gel electrophoresis. Requirements for acceptable gDNA were as follows: total mass ≥1μg, absorbance ratio A260/A280 at ≥ 1.80 and ≤ 2.0. Then a pool containing upstream and downstream oligonucleotides specific to the targeted regions of interest was hybrids to the gDNA samples. Then gDNA was fragmented using a Bioruptor Interrupt instrument (Diagenode, Belgium) and quantitative detection was performed to ensure average fragment size of 150bp to 250bp. Fragmentation was followed by end repair, dA tailing, and sequencing adaptor ligation by ABI 9700 PCR instrument (ABI, USA). The adapter-ligated DNA was amplified by selective, limited-cycle PCR for 5 cycles and then quantitatively analyzed using Qubit dsDNA HS Assay Kit (Invitrogen, USA). Prepared library (750ng) was hybridized with 11μl hybridization block (Allwegene, China), 20μl hybridization buffer (Allwegene, China) and a mix of 5μl RNase block (Invitrogen, USA) and 2μl Probe (Allwegene, China) for overnight (at least 8-16h) at 65°C. The hybridized products were mixed with 200μl nabeads MyOne Streptavidin T1 magnetic beads (Invitrogen, USA) for 30 min at room temperature. After two times of washing by wash buffer (Allwegene, China), the mixture was amplified for 16 PCR cycles and quantitatively assessed using Qubit dsDNA HS Assay Kit (Invitrogen, USA). Captured libraries were denatured and loaded onto an Illumina cBot instrument at 12 to 16pmol/L for cluster generation according to the manufacturer’s instructions. Up to 20 WUCaMP libraries were sequenced per HiSeq lane. A PhiX control (Illumina) was added to lane 8 of each flowcell.

### Analysis of NGS data

Sequencing data, such as the number of altered chromosomes, genomic alternation information and the determination of the depth of sequencing coverage, were analyzed. All analyzed were based on the human reference sequence UCSC build hg19 (NCBI build 37.2) using the Burrows-Wheeler Aligner (BWA) [44]. Local alignment and duplication removal were completed by the application of the Genome Analysis Tool Kit (GATK) and Picard software. Detection of SNPs was performed using dbSNP 132. Damaging or deleterious SNPs were predicted using the Gemini software, and prediction tools, including sorting intolerant from tolerant (SIFT) and polymorphism phenotyping (PolyPhen) were used for the analysis of all human non-synonymous SNPs. In addition, putative somatic variant calls were detected with two separate programs, MuTect 1.1.5 and VarScan 2.3.6 softwares, pairing each sample with its matched blood.

### Statistical analysis

Hardy-Weinberg equilibrium (HWE) was analyzed using gene frequencies obtained by a single gene counting. Chi-square test was used to compare observed and expected values. Genotype association analysis was performed using dominant model (minor allele homozygotes plus heterozygotes vs. major allele homozygotes), recessive (minor allele homozygotes vs. heterozygotes plus major homozygotes), additive model (major homozygotes vs. heterozygotes vs. minor homozygotes), HET model (major homozygotes vs. heterozygotes) and HOM model (major homozygotes vs. minor homozygotes). Genotypic frequencies comparisons between control and ABMR groups were assessed by the chi-square test. In addition, we explored linkage disequilibrium (LD) blocks using Haploview version 4.2 software. Odds ratios (OR) and 95% confidence intervals (95% CIs) were calculated by SPSS 13.0 software (SPSS Inc., Chicago, IL, USA). *P*<0.05 was considered significant. The OR provides an effect estimate, the value of which less than 1 is considered as a protective effect, whereas the value more than 1 is associated with an increased risk. In addition, the genotypic distributions of the *C3* SNPs in ABMR recipients and in stable subjects were analyzed with logistic regression models adjusted for age, sex and immunosuppressive protocol.

## SUPPLEMENTARY MATERIALS TABLES







## Abbreviations

ABMR: antibody-mediated rejection
HLA: human leukocyte antigens
MHC: major histocompatibility complex
DSA: donor-specific antibodies
PRA: panel reactive antibody
SNP: single nucleotide polymorphisms
HWE: Hardy-Weinberg equilibrium
NGS: next-generation sequencing
AMD: age-related macular degeneration
BD: Behcet’s disease
VKH: Vogt-Koyanagi-Harada syndrome
CNV: copy number variations
DCD: donors of cardiac death
DGF: delayed graft dysfunction
Scr: serum creatinine
CsA: Cyclosporin A
MMF: mycophenolate mofetil
SIFT: sorting intolerant from tolerant
GATK: Genome Analysis Tool Kit
LD: linkage disequilibrium
OR: odds ratios
95%CIs: 95% confidence intervals
