# Supplementary material for: Impact of complement component 3/4/5 single nucleotide polymorphisms on renal transplant recipients with antibody-mediated rejection
Source: Oncotarget. 2017 Oct 10;8(55):94539–53. doi: 10.18632/oncotarget.21788 (PMC5706894; doi:10.18632/oncotarget.21788)
Supplement: Supplementary file 1 [file oncotarget-08-94539-s001.pdf]

Impact of complement component 3/4/5 single nucleotide polymorphisms on renal transplant recipients with antibody-mediated rejection

SUPPLEMENTARY MATERIALS

Supplementary Table 1: Genotype frequencies of *C3* genetic polymorphisms in 199 Chinese subjects

See Supplementary File 1

Supplementary Table 2: Genotype frequencies of *C4* genetic polymorphisms in 199 Chinese subjects

Location/Genotypes	Stable group (n=131)	ABMR group (n=68)
**chr6:31963786**		
CC	121	64
CA	10	4
**chr6:31964228**		
AA	116	59
AG	13	8
GG	2	1
**chr6:31964391**		
CC	130	66
CG	1	2
**chr6:31964584**		
CC	116	58
CT	15	10
**chr6:31994974**		
GG	117	59
GA	14	9
**chr6:31996524**		
CC	98	50
CA	27	18
AA	6	0
**chr6:31996966**		
GG	100	50
GA	29	16
AA	2	2
**chr6:31997321**		
CC	126	66
CT	5	2
**chr6:31997401**		
GG	81	45
GA	49	23
AA	1	0

ABMR, antibody-mediated rejection.

Supplementary Table 3: Genetic distributions of C5 polymorphisms between ABMR and stable group

See Supplementary File 2
