# Supplementary material for: Impact of complement component 3/4/5 single nucleotide polymorphisms on renal transplant recipients with antibody-mediated rejection
Source: Oncotarget. 2017 Oct 10;8(55):94539–53. doi: 10.18632/oncotarget.21788 (PMC5706894; doi:10.18632/oncotarget.21788)
Supplement: Supplementary file 2 [file oncotarget-08-94539-s002.doc]

**Supplementary Table 1: Genotype frequencies of *C3* genetic polymorphisms in 199 Chinese subjects**

| **Genotype** | **Stable group** | **ABMR group** |
| --- | --- | --- |
| **rs17030** |  |  |
| **GG** | 32 | 17 |
| **GA** | 62 | 38 |
| **GG** | 37 | 13 |
| **rs344555** |  |  |
| **TT** | 13 | 4 |
| **TC** | 53 | 35 |
| **CC** | 65 | 29 |
| **rs2277984** |  |  |
| **CC** | 31 | 17 |
| **CT** | 63 | 38 |
| **TT** | 37 | 13 |
| **rs7951** |  |  |
| **GG** | 107 | 56 |
| **GA** | 23 | 11 |
| **GG** | 1 | 1 |
| **rs2241394** |  |  |
| **GG** | 118 | 59 |
| **GC** | 12 | 9 |
| **rs2241393** |  |  |
| **GG** | 127 | 64 |
| **GC** | 0 | 4 |
| **CC** | 4 | 0 |
| **rs7257062** |  |  |
| **CC** | 124 | 63 |
| **CT** | 4 | 3 |
| **TT** | 3 | 2 |
| **rs11569536** |  |  |
| **GG** | 129 | 66 |
| **GA** | 2 | 1 |
| **rs3745568** |  |  |
| **TT** | 114 | 57 |
| **TG** | 17 | 11 |
| **rs3745567** |  |  |
| **CC** | 118 | 58 |
| **CT** | 13 | 10 |
| **rs2287845** |  |  |
| **GG** | 1 | 1 |
| **GA** | 27 | 20 |
| **AA** | 103 | 47 |
| **rs366510** |  |  |
| **GG** | 1 | 1 |
| **GT** | 27 | 20 |
| **TT** | 103 | 47 |
| **rs408290** |  |  |
| **GG** | 96 | 41 |
| **GC** | 11 | 14 |
| **CC** | 24 | 13 |
| **rs2230205** |  |  |
| **CC** | 29 | 12 |
| **CT** | 67 | 47 |
| **TT** | 35 | 9 |
| **rs2230204** |  |  |
| **CC** | 49 | 20 |
| **CT** | 61 | 40 |
| **TT** | 21 | 8 |
| **rs10411506** |  |  |
| **GG** | 29 | 13 |
| **GA** | 68 | 47 |
| **AA** | 34 | 8 |
| **rs2230201** |  |  |
| **CC** | 28 | 12 |
| **CT** | 68 | 46 |
| **TT** | 35 | 10 |
| **rs2250656** |  |  |
| **TT** | 86 | 40 |
| **CT** | 43 | 25 |
| **CC** | 2 | 3 |

ABMR, antibody-mediated rejection.
