# Supplementary material for: Impact of complement component 3/4/5 single nucleotide polymorphisms on renal transplant recipients with antibody-mediated rejection
Source: Oncotarget. 2017 Oct 10;8(55):94539–53. doi: 10.18632/oncotarget.21788 (PMC5706894; doi:10.18632/oncotarget.21788)
Supplement: Supplementary file 3 [file oncotarget-08-94539-s003.doc]

**Supplementary Table 3: Genetic distributions of C5 polymorphisms between ABMR and stable group**

| **Genotype** | **Locations** | **Stable group (n=131)** | **ABMR group (n=68)** |
| --- | --- | --- | --- |
|
| rs76339932 | 123716260 |  |  |
| CC |  | 123 | 64 |
| CT |  | 8 | 4 |
| rs12237774 | 123725971 |  |  |
| CC |  | 90 | 49 |
| CT |  | 38 | 17 |
| TT |  | 3 | 2 |
| rs2300931 | 123726145 |  |  |
| TT |  | 107 | 53 |
| TC |  | 23 | 13 |
| CC |  | 1 | 2 |
| rs10985112 | 123731408 |  |  |
| GG |  | 123 | 64 |
| GA |  | 8 | 4 |
| rs2269066 | 123737018 |  |  |
| CC |  | 88 | 46 |
| CT |  | 38 | 20 |
| TT |  | 5 | 2 |
| rs41260544 | 123744252 |  |  |
| AA |  | 125 | 64 |
| AG |  | 6 | 4 |
| rs117287858 | 123752088 |  |  |
| TT |  | 130 | 66 |
| TC |  | 1 | 2 |
| rs2230212 | 123753514 |  |  |
| CC |  | 110 | 59 |
| CA |  | 21 | 9 |
| rs41311867 | 123753600 |  |  |
| GG |  | 125 | 64 |
| GT |  | 6 | 4 |
| rs187517049 | 123753619 |  |  |
| TT |  | 130 | 66 |
| TC |  | 1 | 2 |
| rs12683026 | 123758632 |  |  |
| AA |  | 125 | 64 |
| AG |  | 6 | 4 |
| rs10985122 | 123770837 |  |  |
| TT |  | 127 | 64 |
| TC |  | 4 | 4 |
| rs41309856 | 123776390 |  |  |
| GG |  | 124 | 64 |
| GC |  | 7 | 4 |
| rs144465545 | 123776397 |  |  |
| TT |  | 129 | 65 |
| TA |  | 2 | 3 |
| rs41309850 | 123778688 |  |  |
| AA |  | 124 | 64 |
| AC |  | 7 | 4 |
| rs181763824 | 123780098 |  |  |
| GG |  | 128 | 67 |
| GC |  | 3 | 1 |
| rs2230214 | 123782299 |  |  |
| GG |  | 124 | 64 |
| GA |  | 7 | 4 |
| rs10985126 | 123783934 |  |  |
| TT |  | 82 | 42 |
| TC |  | 41 | 22 |
| CC |  | 8 | 4 |
| rs10985127 | 123783993 |  |  |
| AA |  | 82 | 42 |
| AG |  | 41 | 22 |
| GG |  | 8 | 4 |
| rs28426093 | 123785545 |  |  |
| GG |  | 120 | 60 |
| GA |  | 11 | 5 |
| AA |  | 0 | 3 |
| rs10818499 | 123800094 |  |  |
| TA |  | 40 | 32 |
| TT |  | 58 | 22 |
| AA |  | 33 | 14 |
| rs17216529 | 123800218 |  |  |
| CC |  | 88 | 48 |
| CT |  | 37 | 18 |
| TT |  | 6 | 2 |
